# A GNAS Gene Mutation’s Independent Expression in the Growth of Colorectal Cancer: A Systematic Review and Meta-Analysis

**DOI:** 10.3390/cancers14225480

**Published:** 2022-11-08

**Authors:** Hafeez Abiola Afolabi, Salzihan Md Salleh, Zaidi Zakaria, Ewe Seng Ch’ng, Siti Norasikin Mohd Nafi, Ahmad Aizat Bin Abdul Aziz, Ahmad Adebayo Irekeola, Yusuf Wada, Sameer Badri Al-Mhanna

**Affiliations:** 1 Department of General Surgery, School of Medical Sciences, Hospital Universiti Sains Malaysia, Health Campus, Universiti Sains Malaysia, Kubang Kerian 16150, Malaysia; 2 Department of Pathology, School of Medical Sciences, Hospital Universiti Sains Malaysia (HUSM), Health Campus, Universiti Sains Malaysia, Kubang Kerian 16150, Malaysia; 3 Department of Pathology, School of Medical Sciences, Universiti Sains Malaysia (USM), Health Campus, Kubang Kerian 16150, Malaysia; 4 Advanced Medical and Dental Institute, Universiti Sains Malaysia USM, Kepala Batas 13200, Malaysia; 5 Department of Human Genome Centre, School of Medical Sciences, Health Campus, Universiti Sains Malaysia, Kubang Kerian 16150, Malaysia; 6 Department of Medical Microbiology and Parasitology, School of Medical Sciences, Health Campus, Universiti Sains Malaysia, Kubang Kerian 16150, Malaysia; 7 Department of Physiology, School of Medical Sciences, Health Campus, Universiti Sains Malaysia, Kubang Kerian 16150, Malaysia

**Keywords:** colorectal cancer, colon cancer, CRC, GNAS gene mutations

## Abstract

**Simple Summary:**

Colorectal cancer progression involves multi-gene aberration of several biomarkers via the downstream regulation of the MARK/ERK cascade. GNAS gene mutation early identification is important as a prognosticating biomarker for colorectal cancer screening and diagnosis. The role of GNAS gene codons R201C and R201H in CRC tumourigenesis under the control of the Gpa33-antigen promoter is almost exclusively expressed in colorectal cancer. A total of 30 studies (10,689 patients) were included in this analysis, the male population was the most of the total participants (6068 of 10,689), amounting to (57%). The occurrence of GNAS mutation in CRC was 4.8%; (*p* < 0.001). Codon R201C (40.7%) and R201H (39.7%) sub-codon mutations were the most identified sub-codon mutations in patients with colorectal cancer respectively.

**Abstract:**

Globally, colorectal carcinoma CRC is the third most common cancer and the third most common reason for cancer-associated mortality in both genders. The GNAS mutations are significantly linked with poor prognosis and failed treatment outcomes in CRC. A systematic review and meta-analysis of multiple studies executed following Preferred Reporting Items for Systematic Reviews and Meta-Analysis (PRISMA) criteria and registered with PROSPERO (registration number: CRD42021256452). The initial search includes a total of 271 publications; however, only 30 studies that merit the eligibility criteria were eventually chosen. Data analysis via OpenMeta Analyst and comprehensive meta-analysis 3.0 (CMA 3.0) software were used to investigate the prevalence of GNAS gene mutation among CRC patients. The meta-analysis consisted of 10,689 participants with most being males 6068/10,689 (56.8%). Overall, prevalence of GNAS mutations was 4.8% (95% CI: 3.1–7.3) with I^2^ = 94.39% and (*p* < 0.001). In 11/30 studies, the frequency of GNAS gene mutations was majorly in codons R201C [40.7% (95% CI: 29.2–53.2%)] and in codon R201H [39.7% (95% CI = 27.1–53.8)]. Overall prevalence of GNAS mutations was highest among the male gender: 53.9% (95% CI: 48.2–59.5%: I^2^ = 94.00%, (*p* < 0.001), tumour location (colon): 50.5% (95% CI: 33.2–67.6%: I^2^ = 97.93%, (*p* < 0.001), tumour grade (Well): 57.5% (95% CI: 32.4–79.2%: I^2^ = 98.10%, (*p* < 0.001) and tumour late stage: 67.9% (95% CI: 49.7–84.3%: I^2^ = 98.%, (*p* < 0.001). When stratified according to study location, a higher prevalence was observed in Japan (26.8%) while Italy has the lowest (0.4%). Overall prevalence of GNAS gene mutations was 4.8% with codons R201C and R201H being the most mutated, and the results conformed with numerous published studies on GNAS mutation.

## 1. Introduction

Worldwide, colorectal cancer (CRC) is a foremost contributor to cancer-related death annually and continues to pose a significant challenge to the world [1]. With reports of greater than 1.8 million new cases of CRC diagnoses and approximately 0.86 million deaths throughout the globe in 2018 [2], CRC is the third most frequently occurring cancer, and the third most common cause of cancer-associated deaths in both genders [3], representing 10% of all cancer diagnosed yearly [4]. Over the past decade, increasing evidence points to the role of G-protein activating subunit gene mutations in the development of tumours, i.e., CRC [5]. Several proteins, including those that are produced by the genes GNAS, GNAQ, GNA11 and GNA12 bind to G-protein-coupled receptors (GPCRs) and are essential for the transduction of cellular signals. The process for the initiation and progression of CRC stems from the accumulation of several aberrant genetic and epigenetic alterations in the epithelium cells of the colon and rectum [2]. Reports on overexpression of the GNAS gene in cancers and, linked with tumourigenesis metastasis and progression are vast [2]; however, the detailed understanding of the genetic contribution of GNAS mutation in colorectal cancer (CRC) progression remains ambiguous and unclear [2,3,6].

Just as with the KRAS, the GNAS gene mutations are frequently detected in lots of tumour types, detected in about 5% of all sequenced malignant tumours, as well as 4–7% in colorectal cancers (CRCs) [4], 41% in intraductal papillary neoplasms of the pancreas [7] and about 15% in liver cancer [8]. GNAS gene mutations has been altered in 3.21% of all cancers [1]. For years, noteworthy advances in comprehending cancer epigenetics, particularly on aberrant DNA-methylation, were widely investigated^6^ because gene aberration or mutations have long been recognized as key determinants in cancer development. However, there is still a downside to this discovery, which is a limited clue to the GNAS gene role in the epigenetics of CRC diagnosis and progression [6,9]. An additional prominent downside to cancer sequencing research is the restricted statistical power to substantially recognize mutated genes that have a midway or lower rate of recurrence of mutation (e.g., 5% frequency) [2]. Considering the importance and functional contribution of the G-subunits genes in CRC progression, GNAS gene is among the top seven most frequently recognized mutated genes in tumourigenesis, such as in CRC; others include APC, KRAS, TCF7L2, epidermal growth factor receptor (EGFR), insulin-like growth factor receptor (IGF1R) and CASP8 [2]. In this present study, the prevalence of GNAS gene mutations was investigated in the CRC genomic profiling of patients diagnosed with CRC.

Although CRC progression involves multi-gene aberration of several biomarkers, the identification and confirmation of prognostication factors and biomarkers can improve the management as an adjunct to the clinicohistopathology data of the patients [3,10]. How? It is because the G-protein-coupled receptors (GPCRs) are regarded as the broadest and most diversified family of cell surface receptors among the eukaryotes [11,12,13,14]. They are the most prevailing signal-regulating networks in mammalian cells for the regulation of cell growth and hormone regulations [7]. The GPCRs interrelate with the G-proteins, which consist of three subunits heterotrimeric G-proteins, namely the Gα-subunit G_s_α, the Gβ-subunit G_s_β and the Gγ-subunit G_s_γ. The α-subunits of the G proteins are further classified into four subfamilies namely G_i_, G_s_, G_12/13_ and G_q_. In humans, G_s_α is encoded by the GNAS complex locus and binds to the guanine nucleotide-binding proteins (heterotrimeric G-proteins), which ultimately leads to a physiological response, usually via the downstream regulation of gene transcription (transmembrane signal transduction). However, when gene mutation such as Missense mutations, nonsense mutations, silent mutations and frameshift insertions occurs, this plays a critical role in promoting cancer cell growth and oncogenic transformation, such as in colorectal cancer CRC. Moreover, these mutations occurring at codon 201 of GNAS activate the adenylate cyclase gene and lead to constitutive cAMP signalling and metastasis [1]. GNAS genes are mutated at a significant frequency in colorectal cancer (CRC). The role of GNAS R201C and R201H in CRC tumourigenesis under the control of the Gpa33-antigen promoter is almost exclusively expressed in colorectal cancer. R201C and R201H activating mutation of GNAS causes augmentation of both the Wnt and ERK1/2 MAPK cascade: together they account for a massive 70–80% of GNAS mutation. Mosaicism of the human GNAS sub codons mutation suggested that GNASR201H and or R201C germ-line transmission may cause embryonic lethality. Through this study, the authors aim to determine the global prevalence of GNAS gene mutation in patient diagnosed with colorectal cancer.

## 2. Materials and Methods

This is a systematic review and meta-analysis comprising several types of research and available studies performed in compliance with the procedures stipulated by the Preferred Reporting Items for Systematic Reviews and Meta-Analysis (PRISMA, Table S1), and the study protocol was registered with PROSPERO with the registration number: CRD42021256452: 10 June 2022 (https://www.crd.york.ac.uk/prospero/#myprospero) [8,15].

### 2.1. Literature Search and Selection Criteria

In this present research, several published papers were re-acquired from five main electronic databases (Web of Science WOS, Medline, Google Scholar, Scopus and ScienceDirect). To ascertain the fulfilment of the aim of the study, the eligible studies were searched and vetted using comprehensive and relevant keywords: (“colon cancer” OR “colorectal cancer” OR “metastatic colorectal carcinoma” OR “metastatic colon cancer” OR “metastatic colorectal cancer” OR “CRC” OR “Rectum”) and (“GNAS” OR “GNA” OR “c-GNAS” OR “cGNA”).

**Figure 1:**
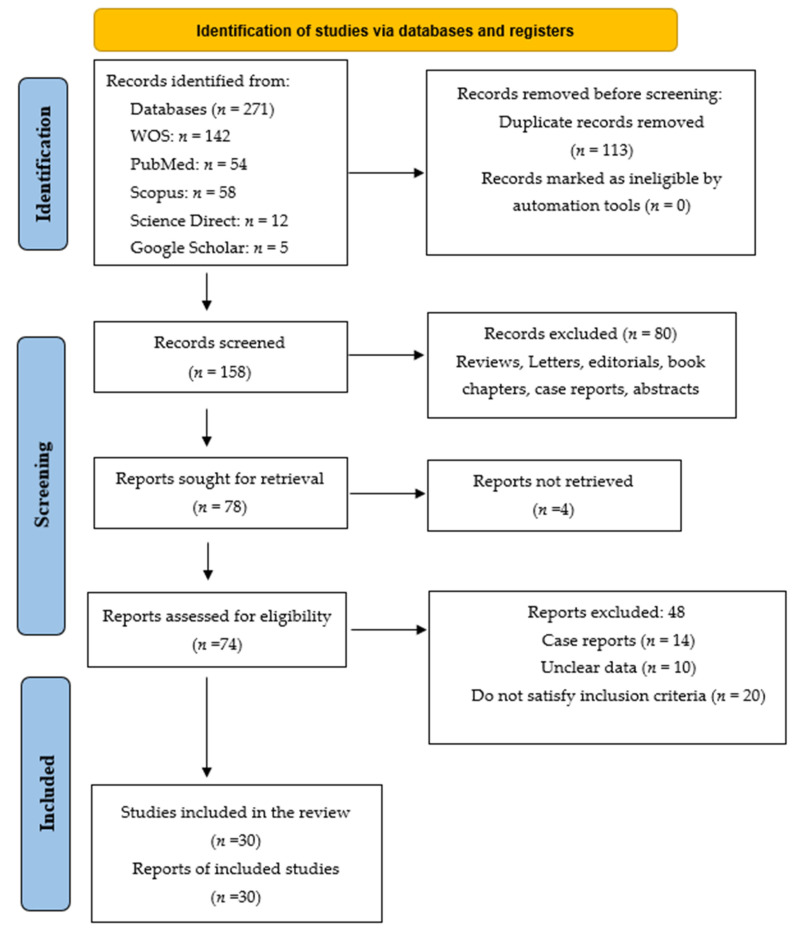
Summary of article identification and selection process.

Detailed comprehensive strategies employed in this study are provided in the Search Strategic File (Text S1). A thorough search for the most pertinent studies was accomplished by scouring through titles, keywords and abstracts of a variety of papers. The preliminary search included 271 articles (Figure 1) that were carried out on the 9^th^ of May 2022 via Mendeley software. The references of all included studies were exported to the software, following which duplicates were then removed. The inclusion criteria selected for use in this meta-analysis study include cross-sectional, cohort or case series performed to determine the frequency of GNAS gene mutation in colorectal cancer patients reported in Fresh Frozen, Formalin-Fixed Paraffin-Embedded FFPE or biopsied colorectal cancer specimens. Moreover, GNAS gene mutation articles consisting of more than one sample size as well as all associated papers published at recognized international summits were considered. No restriction is set on methods for demonstrating gene mutations. The exclusion criteria entail (1) research not related to frequency of GNAS gene mutation, (2) research that examined just one of either codon R201C or R201H of GNAS gene mutation, (3) reviews and case reports and (4) GNAS gene mutations that are linked to cell lines and animal research [16]. All authors participated in the study screening, selection and assessment criteria. Two authors (H.A.A. and S.M.S.) independently screened the publications based on the study’s title and abstract. Any dissonances during the screening process were solved by dialogue with other supporting authors in the study. 

### 2.2. Data Extraction and Quality Assessment

The data extraction was performed on an Excel spreadsheet. Two reviewers (H.A. and S.S) independently examined the titles and abstracts and extracted pertinent information needed, i.e., study identity, year of study publication, period and design, gender and report of GNAS gene mutation prevalence reported amongst patients with the diagnosis of colorectal cancer. Any discrepancies were addressed via dialogue with a third reviewer (A.A.I.) to avoid any bias, and any incongruities were sorted out via discussion involving other reviewers to avert bias. The quality of the methodological approach for the studies included was appraised independently by two authors (H.A. and Y.W.) via the Joanna Briggs Institute (JBI) critical appraisal checklist for prevalence data [17] (Table S2). A score of 1 for “Yes” and 0 for other parameters was allotted to obtain a sum quality score that ranges between 0 and 9. Studies with a final score of 7–9 were chosen to be of desirable quality. The studies within the latter acceptable score range were included in the data extraction phase for the meta-analysis.

### 2.3. Data Synthesis and Analysis

The data analysis was performed using OpenMeta Analyst and comprehensive meta-analysis 3.0 (CMA 3.0) software [18]. The prevalence of GNAS gene mutation amongst colorectal cancer patients was computed, and data analysis on subgroup variables was also performed on tumour location, gender, tumour stage, study year and tumour grade. A random effect model using the DerSimonian–Laird method of the meta-analysis was used to obtain the pooled estimates of the recorded GNAS gene mutation cases. Moreover, to uphold the quality and soundness of the study, probable publication bias was carefully vetted by generating a funnel plot. The asymmetry of the funnel plot was further examined via Egger’s regression test [11]. Cochran’s Q test and quantification using I^2^ statistics were used to determine the study-level heterogeneity, with the values of I^2^ at 25%, 50% and 75% designated as “Low”, “Moderate” and “High” heterogeneity, respectively. In all tests, a *p*-value of less than 0.001 was classified as statistically significant.

## 3. Result

To make the result section concise and precise, the result presentation was written in subsections with each ascribed subheading to illustrate the experiment findings and interpretation as well as the inferential conclusion carved out from the outcomes.

### 3.1. Search Results and Study Selection

This present study involves a total of 271 articles obtained by exploring five electronic databases. After removing the duplicates and studies that do not conform with the inclusion criteria, 158 studies were remaining for screening through titles and abstracts, thus leading to the exclusion of another 80 studies. Upon more rigorous vetting of the manuscripts, another 48 studies with incomplete records and those that satisfied the exclusion criteria were removed (illustrated in Figure 1 above). Finally, a total of 30 studies were considered eligible to be included in the meta-analysis. Among the eligible 30 studies selected for this meta-analysis report on GNAS gene mutation, 11 studies reported on the GNAS codon R201C and R201H, both of which are considered the most identified codons in GNAS gene mutations. Thus, a total of 30 studies were selected for this meta-analysis.

### 3.2. Characteristics of the Eligible Studies

Table 1 below was designed to show the comprehensive characteristics of the included studies on GNAS gene mutation. The meta-analysis study comprises 10,689 sample size; the studies spanned across the globe with the most numbers coming from the United States. Overall, the male population comprised most of the total participants (6068 of 10,689), amounting to (57%).

### 3.3. Prevalence of GNAS Mutations in CRC Patients

**Figure 2:**
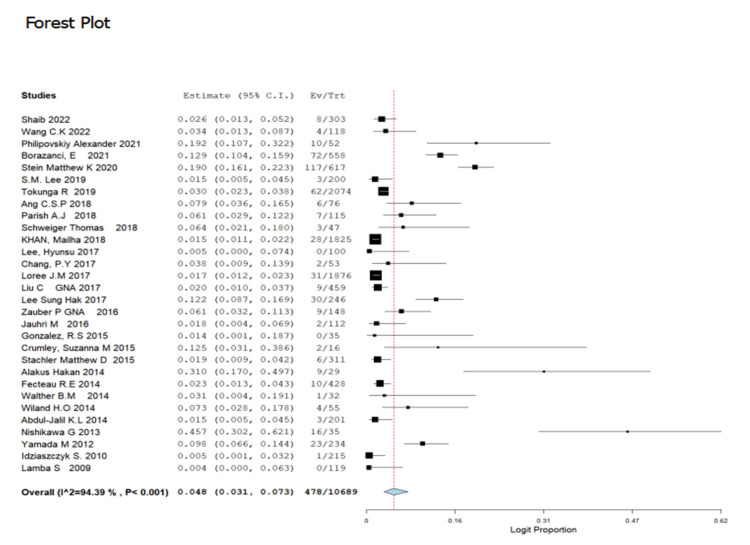
Forest plot for the prevalence of GNAS mutation in CRC patients [1,6,12,14,20,21,22,23,24,25,26,27,28,29,30,31,32,33,34,35,36,37,38,39,40,41,42,43,44,45].

**Figure 3:**
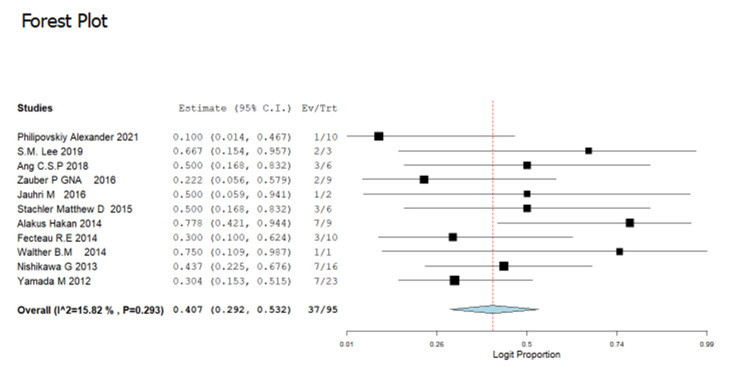
Forest plot for GNAS codon R201C in CRC patients [1,6,14,22,24,33,36,37,38,39,43].

**Figure 4:**
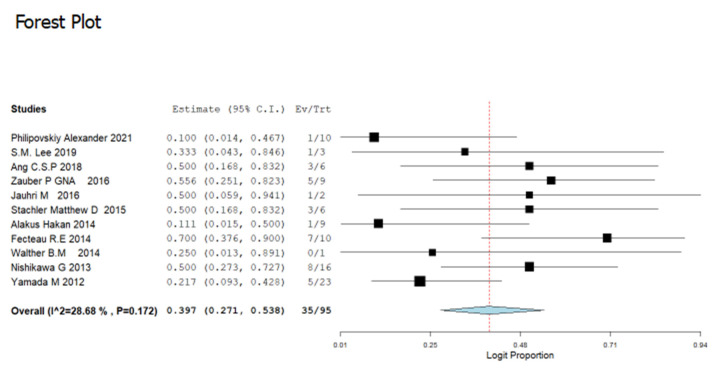
Forest plot for GNAS codon R201H in CRC patients [1,6,14,22,24,33,36,37,38,39,42].

The prevalence of GNAS gene mutation depicted in the 30 selected studies incorporated in the meta-analysis consist of a total of 10,689 patients. Amongst the studies, the greatest frequency of GNAS gene mutations was reported by [42] at a rate of 45.7% (95% CI:30.2–62.1%) while the lowest frequency of GNAS gene mutations was reported by [44]: 0.4% (95% CI: 0.00–6.3%). Employing the random effect model, the overall prevalence of GNAS gene mutations was 4.8% (95% CI: 3.1–7.3) with I^2^ = 94.39% and (*p* < 0.001) (Figure 2). Furthermore, 11 out of the 30 included studies reports on the frequency of GNAS codon mutations reported codons R201C and R201H as the most prevailing. The prevalence of the mutated codons across all GNAS mutations is presented in Figure 3 and Figure 4. Codon R201C and R201H mutations were found in the populations to be 40.7% (95% CI: 29.2–53.5) and 39.7% (95% CI = 27.1–53.8), respectively (Figure 3 and Figure 4).

### 3.4. Prevalence of GNAS Gene Mutation in Colorectal Cancer Stratified by Study Location and Period of Study

To investigate the prevalence of GNAS gene mutation in CRC patients from various regions, a subgroup meta-analysis was carried out. There was available data for 12 locations from the included studies, with the highest number of studies recorded in the United States US (*n*:16) (Table 2; Figure S1). The country of Japan recorded the highest prevalence rate at 26.8% (95% CI: 0.083–0.620), while Italy recorded the lowest prevalence at 0.4% (95% CI: 0.007–0.016) (Table 2; Figure S1).

On the gender predilection of study, the male gender (6,068 of 10,689) had the highest prevalence of GNAS gene mutation 57% (95% CI: 0.482–0.595), respectively; *p* < 0.001) when compared to the female counterpart 43% (95% CI: 0.378–0.492), respectively; *p* < 0.001) (Table 2; Figures S2 and S3).

In the tumour stage, GNAS gene mutation was recorded highest in the late stage at 67.9% (95% CI: 0.497–0.843) than the early stage, while in tumour location, the colon has the highest GNAS gene mutation of 50.5% (95% CI: 0.332–0.676) for the tumour located in the colon. On the grading of GNAS gene mutation in CRC, “Well graded” recorded the highest GNAS gene mutation of 57.5% (95% CI: 0.324–0.792) while the “Moderately graded” has the least prevalence value of 10.7% (95% CI: 0.033–0.296) (Table 2; Figure S4, S5, S6, S7, S8 and S9 respectively).

### 3.5. Analyses of Sensitivity and Publication Bias

**Figure 5:**
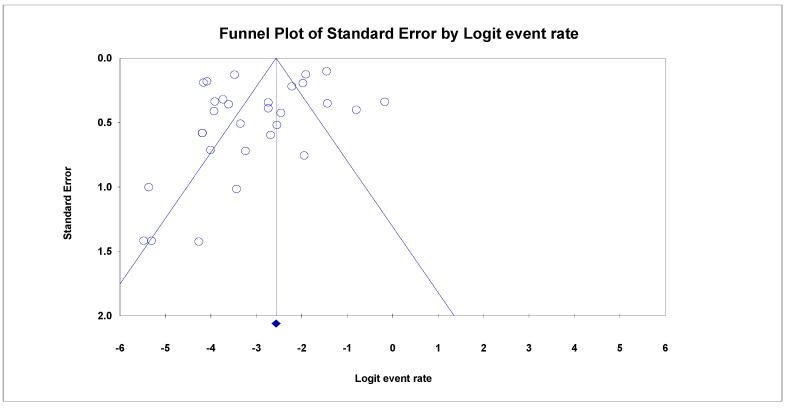
GNAS Funnel Plot Funnel plot showing no significant publication bias (Egger’s *p* = 0.12281).

A funnel plot of random effects was created to look for signs of publication bias in papers reporting GNAS gene mutations among patients with CRC (Figure 5). However, the GNAS mutant studies lacked glaring indications of publication bias.

## 4. Discussion

A third of all carcinosis is understood to be caused by mutations in the RAS family of genes, particularly the downstream activation of the heterotrimeric G-protein α subunits (G_s_α), probably due to its overwhelming effects on the stimulation of Ras, basically turning it on and off. Nonetheless, the occurrence of these mutations differs based on the cancer type, approximately 5–7% in colorectal cancer [46], 10–15% in hepatocellular cancer [9,13] and 21% in pancreatic carcinoma [5]. Due to the lack of early occurring signs with long-term recesses linked with the early onset of organ metastases in CRC, only a small number of patients with the disease would be opportune to receive curative surgery at the time of consultation in the healthcare facility [46]. Additionally, because CRC grows gradually over time from the constellation of genetic anomalies, the risk of recurrence and mortality from colorectal cancer is strongly correlated with the stage of the disease upon diagnosis; hence the need for prognosis predicting biomarker [44,47,48]. Even though there has been a substantial advancement in the treatment of CRC using cytotoxic drugs, such as monoclonal antibodies to targeted therapy such as on the EGF receptor [49], the GNAS gene mutation is still regarded as a prominent contributor to treatment failure in cancer management and, hence, poor prognosis.

Representing 4.2% of all new cases of cancer [50], CRC is the third most prevailing and third most common cancer-related death worldwide [9,50]. In 2018 alone, CRC accounts for over 880,000 deaths and 1.9 million new cases [51]. However, there are considerable regional differences in the incidence and mortality rates of CRC, in this analysis, 30 studies were eventually selected from an initial overall of 271 articles to determine the prevalence of GNAS gene mutation among CRC patients globally. In the course of this study, some related 48 articles reporting on GNAS gene mutation in CRC were found, but they were excluded because they did not meet the inclusion criteria for this study. These glut of papers uncovered spanned almost every nook and cranny of the globe; ref. [43] reported the first occurrence of GNAS mutation in CRC in the United Kingdom, [39] confirmed the prevalence case of GNAS in German patients in Europe while [52] and [53] were conducted in Africa. Collectively, these show the various global prevalence of GNAS mutations in CRC.

In the present study, the prevalence of GNAS gene mutations was examined in 30 studies involving 10,689 patients diagnosed with CRC from different countries around the globe. The overall prevalence of GNAS gene mutations was 4.8% (95% CI: 3.1–7.3) with I [2] = 94.39%, *p* < 0.001). GNAS gene mutation is a comprehensively investigated mutation in many cancers probably because it functions as the most common cancer-initiating mutation across the heterotrimeric G-proteins, the Gα-subunit cascade of the MAPK/ERK pathway. It is perhaps also because it is an active oncogene found in several tumour types in various percentages, i.e., 15–21% in the intraductal pancreas and liver cancer [47] and 3.5–7% of CRC cases globally [44,48]. The findings of the latter investigations substantiate our study’s outcome that about 4% of CRC patients have GNAS gene mutations. This prevalence rate was analogous to figures recorded in Spain (4.7%) [54], the US (5.4%) [1], Taiwan (4.0%) [29], the United Kingdom (1.0%) [44] and India (3.2%) [55] though GNAS mutation prevalence was revealed to somewhat differ or not be present from some available data from Korea [30], Tokyo [56], Turkey [57] and the UAE [58]. The latter contrasts could be related to multiple reasons ranging from a racial predilection to lifestyle, phase and route of specimen collection and geographical settings. The prevalence of the GNAS gene mutation was highest among patients screened in Japan (26.8%) and Norway (12.9%), respectively, and lowest in Italy (0.4%) and the United Kingdom (0.5%).

It is well known that the incidence of genomic and epigenetic alterations leading to tumourigenesis is dynamic [59,60]. In the present study, the majority of the selected patients were adults, with the majority of them being over 50 years old, suggesting that GNAS gene mutation dominates in the adult population. This outcome was exactly as expected given that ageing has historically and medically been linked to a higher risk of CRC in various studies [61]. Moreover, the male patients were found to have a higher predisposition to CRC at a higher rate (57%) than their female counterparts. This information is comparable with results from other published articles conducted worldwide [62,63] and indicates the significance of gender roles in the prevalence of CRC. The majority of the mean ages registered by the studies were in their fifth or sixth decade of life. There are numerous explanations for these variations, ranging from dietary preferences to lifestyle changes that synergically combined to refashion our body’s bio-genetic makeup [64]. Moreso, clinical presentations occur gradually over a lengthy period or with advancing age, then lead to a decrease health status that eventually allows worsening symptoms of the disease; hence the late stage and the older age predilection to CRC.

Further, in the tumour stages, the “late stage” (stages 3 & 4) had more GNAS gene mutation (68%) than the early stage (27%). This could be attributed to differences in the consultation period and tumour stages at the time of patient enlistment for the involved studies. 

It is almost true that most CRC patients would consult at a later stage of the disease [65]; this may explain why the colon (50.5%) rather than the rectum (21.0%) was the most common primary site of the tumour reported in this study, and this finding is consistent with numerous released studies [6,55]. However, most of the selected studies classified the tumour location as either in the colon or rectum, therefore giving the colon a bigger proportion [66,67]. Nonetheless, the rectum accounted for 21%, which, if all studies had classified the location based on the various sections of the colon, i.e., transverse, ascending, descending, sigmoid and rectum, respectively, means it is very likely that the rectum may account for the highest proportion.

GNAS codon 201 aberrations are particularly frequently detected in cancer, especially as they lead to fundamental activation of G_s_α and autonomous cyclic-AMP release. It is worth mentioning in the findings the two most identified codons of GNAS gene mutation: R201C and R201H reported by 11 of the 30 studies, the majority of GNAS mutations were recorded in codon R201C; 40.7% (95% CI: 29.2–53.2%) and codon R201H; 39.7% (95% CI = 27.1–53.8), both codons have almost similar occurrence rates. The latter findings were as reported in previous studies [1,38]. For example, in US research, 5.0% of people had GNAS mutations, with 83% of mutations in codons R201H [40]. Another study published in the Republic of Korea [22] found that GNAS mutations in codons 201 were found in 91.3%. A similar study in Australia reported that although there was synergistic detection of KRAS along with GNAS mutation in CRC, 87% of the GNAS codon was majorly in codons R201C and R201H [33]. However, some studies reported no detections of GNAS mutation in some CRC research [28,68]. Only two studies on colorectal cancer patients in our study reported other GNAS codons: R201S and Q227H mutations, respectively [1,37]. These former two codons are the most commonly detected GNAS codon in CRC and their presence denotes a blueprint in the therapy approach. This is as stated in most GNAS gene mutations carried out by various studies in patients diagnosed with CRC [34,69].

Although the GNAS gene is also part of the MAPK/ERK pathway (or Ras-Raf-MEK-ERK pathway) family just like KRAS, that activation of mutation in the GNAS gene fosters tumourigenesis by activating the Wnt/β-catenin pathway or the ERK1/2 MAPK pathway; however, their mutations are less frequent than the KRAS gene mutations [70]. However, the whole genomic analysis showed that aberrations affecting G-proteins and GPCRs are more frequently occurring than previously assumed in transmuted cells. GNAS-activating mutations are frequently exclusive with CRC progression, accounting for approximately 5–10% of mCRC cases and are associated with poor prognostics, especially in the late stages [71]. This mutation causes a constant stimulation of the mitogen-activating protein kinase MAPK-pathway, which controls the transcriptase activity of regulatory genes in the cell cycle by modulating cell growth stimuli [65]. Genetic homogeneity could be used as an explanation for the prevalence of similarity, as well as patients’ lifestyles and diets.

By classifying cancer types including colorectal cancer and subtypes, i.e., codons according to their genetic make-up via sequencing machines such as Next Generation Sequencing NGS machine, which avails cancer genomics an advanced precision medical therapy. This genetic classification of CRC can offer patients a more accurate diagnosis and, consequently, a more specialized course of treatment. This is because early identification of the mutations and sub-codon mutation will increase sensitivity to finding low-frequency variations or aberrations. Moreover, ensuring quicker turnaround for large numbers of the patient sample with a thorough screening of broad genomic coverage. The main goal of identifying mutation sequencing is to gather medically useful information for the future treatment of various types of cancer. Even in asymptomatic individuals, genomic sequencing can reveal genetic variations that either cause disease or raise the risk of disease development. The outcomes of this study will enable medical professionals to simultaneously evaluate several cancer-related genes. After a patient has had a biopsy or had their tumour surgically removed, tissue from the tumour can be sequenced using machines such as next-generation technology.

The present study has several benefits and strengths. To the best of the authors’ knowledge, this is the first systematic review and meta-analysis to report on the prevalence of GNAS gene mutation in patients with CRC. Additionally, a very comprehensive search strategy ensures that intricate, all-encompassing papers are included for analysis in this study, resulting in a very large population size of 10,689. This latter approach promotes a high level of confidence in the results because the included research had excellent methodology designs. 

This analysis did have some limitations, many of which were related to the data from the included studies’ literature, including the small sample size, incomplete reports on sex, mean age, period of study conduct, tumour differentiation and location and, finally, the fact that mutation screening was restricted to two sub-codons. Some of the studies analysed in this meta-analysis did not report all these features or characteristics, which accounts for some of the heterogeneity observed in the research.

## 5. Conclusions

The prevalence of GNAS mutations in CRC patients was illustrated in this systematic review and meta-analysis, which, to our knowledge, is the first report on the subject. Despite a few drawbacks, the meta-analysis produced striking results. The total prevalence of GNAS gene mutation is 4.8% and differs country-wise. Furthermore, it was found that the prevalence of these mutations noted in our research was consistent with other studies’ findings when the results of our investigation were compared to those of other studies.

## Figures and Tables

**Table 1:** Major characteristics of the prevalence of KRAS screening studies included in the meta-analysis.

Nr.	Author	Year	Location	Male n (%)	Age *	Sample size	Tumour Stage (Early Stage 1&2)	Tumour Stage (Late-Stage 3&4)	Tumour Location (Colon)	Tumour Location (Rectum)	Tumour Grade (Poor)	Tumour Grade (Moderate)	Tumour Grade (Well)	Method	Total GNAS Mutation (%)	GNAQ (%)	GNA11 (%)
1	Shaib et al. [12].	2022	USA	44	56.8 (54–83)	303	NR	NR	NR	NR	NR	NR	NR	NGS-sequencing	2.6	NR	NR
2	Wang et al. [19]	2022	USA	64.4	52 (19–88)	118	22	78	NR	NR	NR	NR	NR	Sanger sequencing	3.4	NR	NR
3	Philipovskiy et al. [14]	2021	USA	69.2	58.67 ± 10.64	52	NR	52	NR	NR	NR	NR	NR	sequencing	19.2	NR	NR
4	Borazanci et al. [20]	2021	Norway	40.5	56 (20–88)	558	NR	NR	NR	NR	NR	NR	NR	NGS-sequencing	12.9	NR	NR
5	Stein et al. [21]	2020	USA	55	59 (16–91)	617	NR	NR	421	147	7.9	49	25	sequencing	19	NR	NR
6	Lee, S.M. et al. [22]	2019	South Korea	54.1	58 (20–80)	200	NR	NR	NR	NR	NR	NR	NR	sequencing	1.5	NR	NR
7	Tokunaga et al. [23]	2019	USA	1435	NR	2074	NR	NR	NR	NR	NR	NR	NR	NGS-sequencing	3	NR	NR
8	Ang et al. [24]	2018	USA	40.8	53.4 (23.6–82.8)	76	8	92	NR	NR	44.7	11.8	NR	sequencing	7.9	NR	NR
9	Parish et al. [25]	2018	USA	60.9	56.1 (1.0–95.1)	115	NR	NR	NR	NR	NR	NR	NR	sequencing	6.1	NR	NR
10	Schweiger et al. [26]	2018	Austria	55.3	63(44–83)	47	34	61.7	59.6	40.4	NR	NR	NR	sequencing	6.4	11	19.1
11	Khan et al. [27]	2018	USA	56.7	55.2 (19.1–91.8)	1825	35.4	64.6	NR	NR	NR	NR	NR	sequencing	1.5	NR	NR
12	Lee, H. et al. [28]	2017	South Korea	60	NR	100	NR	NR	NR	NR	NR	NR	NR	sequencing	0.5	NR	NR
13	Chang et al. [29]	2017	Taiwan	75	58 (26–75)	53	NR	NR	81	19	NR	NR	NR	sequencing	3.8	NR	NR
14	Loree et al. [30]	2017	USA	56	55(46–63)	1876	78.2	3	77.5	22.5	NR	NR	NR	NGS-sequencing	1.7	NR	NR
15	Liu, C. et al. [31]	2017	Australia	53.8	68.3 ± 13.5	459	NR	NR	NR	NR	NR	NR	NR	Sanger sequencing	2	NR	NR
16	Lee, S.H. et al. [32]	2017	South Korea	150	NR	246	NR	NR	NR	NR	NR	NR	NR	W.E. Sequencing	12.2	0.9	NR
17	Zauber, M. et al. [1]	2016	USA	30	69 (24–95)	148	52	44.6	NR	NR	NR	NR	NR	sequencing	6.1	NR	NR
18	Jauhri et al. [33]	2016	India	70	NR	112	NR	NR	NR	NR	NR	NR	NR	NGS-sequencing	1	0.9	NR
19	Gonzalez et al. [34]	2015	USA	40	69 (27–89)	35	31	69	69	19	3	17	NR	Sanger sequencing	1.4	NR	NR
20	Crumley et al. [35]	2015	USA	56.3	57 (21–85)	16	0	100	0	100	19	0	NR	NGS-sequencing	12.5	NR	NR
21	Stachler et al. [36]	2015	USA	61	56.9 (21–89)	311	18	68.5	72	27.6	30.2	0	3.9	sequencing	1.9	NR	NR
22	H Alakus et al. [37]	2014	USA	20	54 (22–90)	29	NR	NR	NR	NR	NR	NR	NR	sequencing	3.1	NR	NR
23	Fecteau et al. [38]	2014	USA	49.5	NR	428	34.3	65.6	NR	NR	NR	NR	NR	pyrosequencing	2.3	NR	NR
24	B M Walther et al. [39]	2014	Germany	20	77 (58–85)	32	NR	NR	NR	NR	NR	NR	NR	sequencing	3.1	NR	NR
25	Wiland IV et al. [40]	2014	USA	47	60 (38–82)	55	NR	NR	45.5	55.5	NR	NR	NR	sequencing	7.3	NR	NR
26	Abdul-Jalil et al. [41]	2014	Ireland	70	63 (38–80)	201	9	87	NR	NR	18	12	4	NGS-sequencing	1.5	NR	NR
27	Nishikawa et al. [42]	2013	Japan	20	56 (18–80)	35	NR	NR	NR	NR	NR	NR	NR	Sequencing	45.7	NR	NR
28	M Yamada et al. [6]	2012	Japan	140	NR	234	NR	NR	NR	NR	NR	NR	NR	Sequencing	9.8	NR	NR
29	Idziaszczyk W et al. [43]	2010	UK	130	NR	215	NR	NR	NR	NR	NR	NR	NR	sequencing	0.5	NR	NR
30	S Lamba et al. [44]	2009	Italy	70	NR	119	NR	NR	NR	NR	NR	NR	NR	NGS-sequencing	0.4	NR	NR

N: Number, NR: Not reported, * Age is presented in years [(mean + SD/median (range/IQR)/range, HRMS: High resolution melting (HRM)-sequencing, HRMA/P: High resolution melting assay/pyrosequencing, PNAM/PCR and PNAM/PCR/S: Peptide Nucleic Acid-mediated Polymerase Chain Reaction/Sequencing, IHC: immunohistochemistry; W.E.S Whole Exome Sequencing.

**Table 2:** Subgroup analysis. Prevalence of GNAS of patients with colorectal cancer stratified by study location of study.

Subgroup	No of Studies	Prevalence (%)	95% CI	I^2^ (%)	Q	Heterogeneity Test	
						DF	*p*
Study Location							
USA	16	4.8	0.033–0.062	90.74	161.95	15	<0.001
Norway	1	12.9	0.101–0.157	NA	NA	NA	NA
South Korea	3	4.2	0.002–0.086	92.93	28.31	2	<0.001
Austria	1	6.4	0.006–0.134	NA	NA	NA	NA
Taiwan	1	3.8	0.014–0.089	77.04	NA	NA	NA
Australia	1	2	0.007–0.032	NA	NA	NA	NA
India	1	1.8	0.007–0.042	NA	NA	NA	NA
Germany	1	3.1	0.029–0.092	NA	NA	NA	NA
Ireland	1	1.5	0.002–0.032	NA	NA	NA	NA
Japan	2	26.8	0.083–0.620	94.2	17.24	1	<0.001
United Kingdom	1	0.5	0.007–0.016	NA	NA	NA	NA
Italy	1	0.4	0.176–0.306	NA	NA	NA	NA
Overall	30	4.5	0.034–0.056	90.82	315.89	29	<0.001
GNAS Subgroup by Gender of Study Conduct							
Male gender	20	56.9	0.482–0.595	94.84	367.997	19	<0.001
Female gender	20	43.4	0.378–0.492	95.08	386.062	29	<0.001
GNAS Subgroup by Tumour Stage							
Early Tumour Stage (1)	11	27.3	0.152–0.441	98.99	987.069	10	<0.001
Late Tumour Stage (2)	11	67.9	0.497–0.843	98.87	974.316	10	<0.001
GNAS Subgroup by Tumour Location							
Colon	8	50.5	0.332–0.676	97.93	338.303	7	<0.001
Rectum	8	21	0.150–0.287	93.52	108.081	7	<0.001
GNAS Subgroup by Tumour Grading							
Poor	6	18.3	0.091–0.334	95.09	101.748	5	<0.001
Moderate	6	10.7	0.033–0.296	95.99	124.645	5	<0.001
Well	6	57.5	0.324–0.792	98.1	263.622	5	<0.001

## Data Availability

All data accessed and analysed in this study are available in the article and its Supplementary Materials.

## Supplementary Materials

The following supporting information can be downloaded at: https://www.mdpi.com/article/10.3390/cancers14225480/s1, Table S1: GNAS Supplementary PRISMA, Table S2: The quality of the 30 included studies, Figure S1: GNAS mutation by countries, Figure S2: Gnas Mutation By Gender. Male Gender, Figure S3: GNAS Mutation By Gender. Female Gender, Figure S4: Gnas Mutation by Tumour Stages. Early Stage, Figure S5: GNAS mutation by the Late tumour stage, Figure S6: GNAS mutation by the Well tumour grade, Figure S7: GNAS mutation by the Well tumour grade, Figure S8: GNAS mutation by the Well tumour grade, Figure S9: GNAS mutation by the Moderate tumour grade, Figure S10: GNAS mutation by the Poor tumour grade, Text S1: SEARCH STRATEGY.
