# Supplementary material for: A GNAS Gene Mutation’s Independent Expression in the Growth of Colorectal Cancer: A Systematic Review and Meta-Analysis
Source: Cancers (Basel). 2022 Nov 8;14(22):5480. doi: 10.3390/cancers14225480 (PMC9688108; doi:10.3390/cancers14225480)
Supplement: Supplementary file 1 [file cancers-14-05480-s001.zip › Supplementary Figures.pdf]

GNAS MUTATION SUPPLEMENTARY FILES OF FOREST PLOTS

Studies	Estimate (95% C.I.)	Ev/Trt
Shaib	0.026 (0.008, 0.044)	8/303
Wang C.K	0.034 (0.001, 0.067)	4/118
Philipovskiy Alexander	0.192 (0.085, 0.299)	10/52
Stein Matthew K	0.190 (0.159, 0.221)	117/617
Tokunga R check again	0.030 (0.023, 0.037)	62/2074
Ang C.S.P	0.079 (0.018, 0.140)	6/76
Parish A.J	0.061 (0.017, 0.105)	7/115
KHAN, Mailha	0.015 (0.010, 0.021)	28/1825
Loree J.M	0.017 (0.011, 0.022)	31/1876
Zauber P GNA	0.061 (0.022, 0.099)	9/148
Gonzalez, R.S	0.014 (-0.024, 0.052)	0/35
Crumley, Suzanna M	0.125 (-0.037, 0.287)	2/16
Stachler Matthew D	0.019 (0.004, 0.035)	6/311
Alakus Hakan	0.310 (0.142, 0.479)	9/29
Fecteau R.E	0.023 (0.009, 0.038)	10/428
Wiland H.O	0.073 (0.004, 0.141)	4/55
**Subgroup USA (I^2=90.74 % , P=0.000)**	**0.048 (0.033, 0.062)**	**313/8078**
Borazanci, E	0.129 (0.101, 0.157)	72/558
**Subgroup Norway (I^2=NA , P=NA)**	**0.129 (0.101, 0.157)**	**72/558**
S.M. Lee	0.015 (-0.002, 0.032)	3/200
Lee, Hyunsu	0.005 (-0.009, 0.019)	0/100
Lee Sung Hak	0.122 (0.081, 0.163)	30/246
**Subgroup South Korea (I^2=92.93 % , P=0.000)**	**0.042 (-0.002, 0.086)**	**33/546**
Schweiger Thomas	0.064 (-0.006, 0.134)	3/47
**Subgroup Austria (I^2=NA , P=NA)**	**0.064 (-0.006, 0.134)**	**3/47**
Chang, P.Y	0.038 (-0.014, 0.089)	2/53
**Subgroup Taiwan (I^2=NA , P=NA)**	**0.038 (-0.014, 0.089)**	**2/53**
Liu C GNA	0.020 (0.007, 0.032)	9/459
**Subgroup Australia (I^2=NA , P=NA)**	**0.020 (0.007, 0.032)**	**9/459**
Jauhri M	0.018 (-0.007, 0.042)	2/112
**Subgroup India (I^2=NA , P=NA)**	**0.018 (-0.007, 0.042)**	**2/112**
Walther B.M	0.031 (-0.029, 0.092)	1/32
**Subgroup Germany (I^2=NA , P=NA)**	**0.031 (-0.029, 0.092)**	**1/32**
Abdul-Jalil K.L	0.015 (-0.002, 0.032)	3/201
**Subgroup Ireland (I^2=NA , P=NA)**	**0.015 (-0.002, 0.032)**	**3/201**
Nishikawa G	0.457 (0.292, 0.622)	16/35
Yamada M	0.098 (0.060, 0.136)	23/234
**Subgroup Japan (I^2=94.2 % , P=0.000)**	**0.268 (-0.083, 0.620)**	**39/269**
Idziaszczyk S.	0.005 (-0.004, 0.014)	1/215
**Subgroup UK (I^2=NA , P=NA)**	**0.005 (-0.004, 0.014)**	**1/215**
Lamba S	0.004 (-0.007, 0.016)	0/119
**Subgroup Italy (I^2=NA , P=NA)**	**0.004 (-0.007, 0.016)**	**0/119**
**Overall (I^2=90.82 % , P=0.000)**	**0.045 (0.034, 0.056)**	**478/10689**

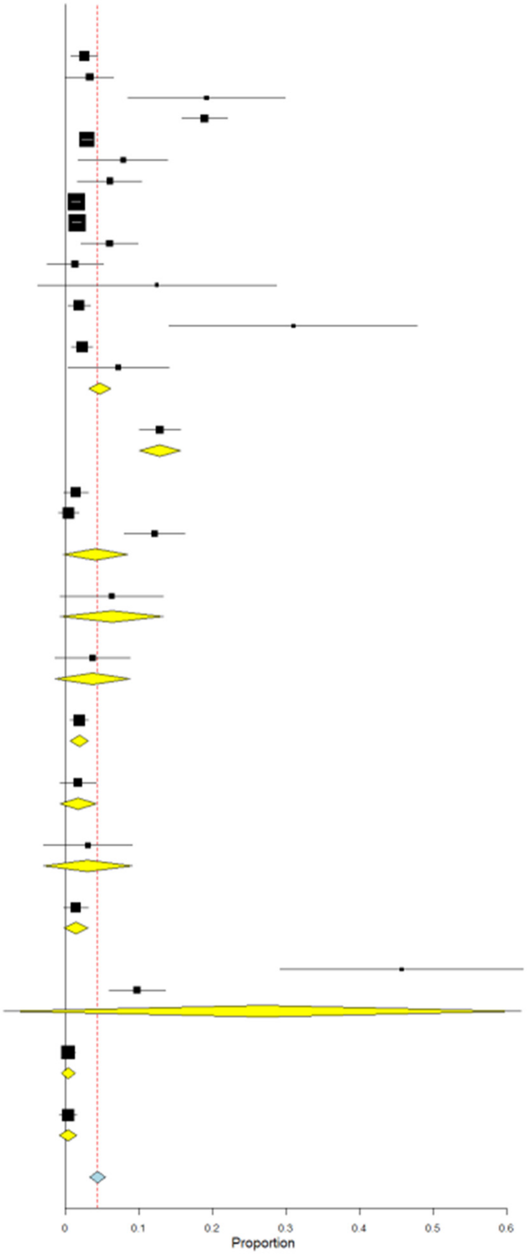

Figure S1. GNAS mutation by countries.

MALE GENDER

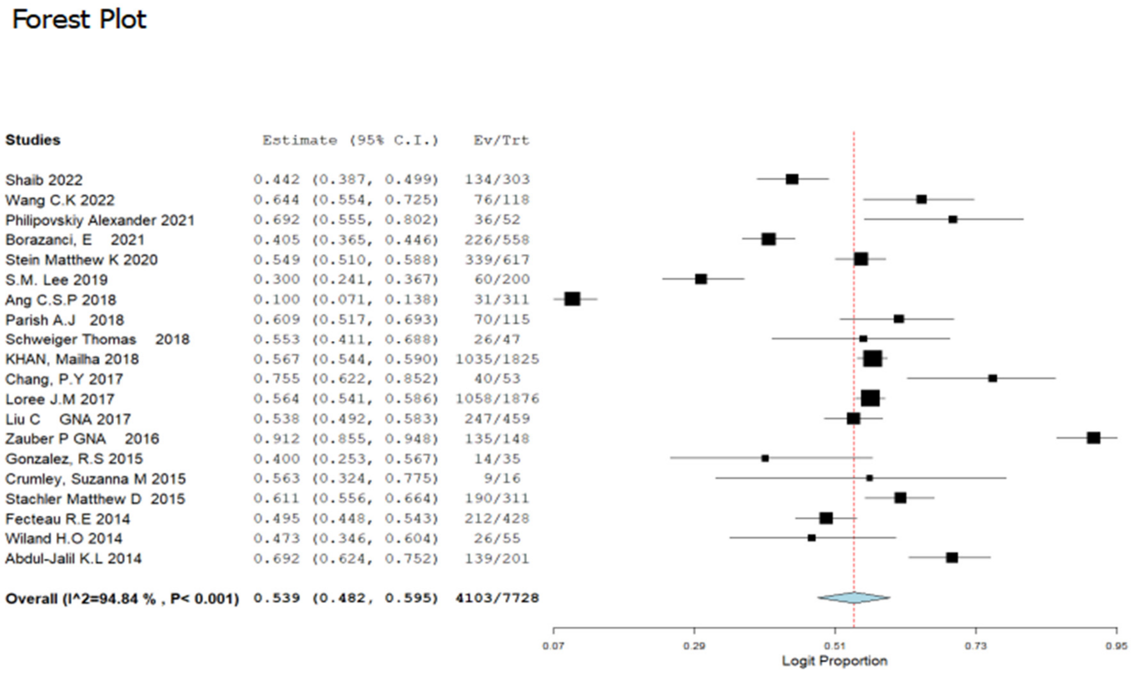

Figure S2. Gnas Mutation By Gender. Male Gender.

FEMALE GENDER

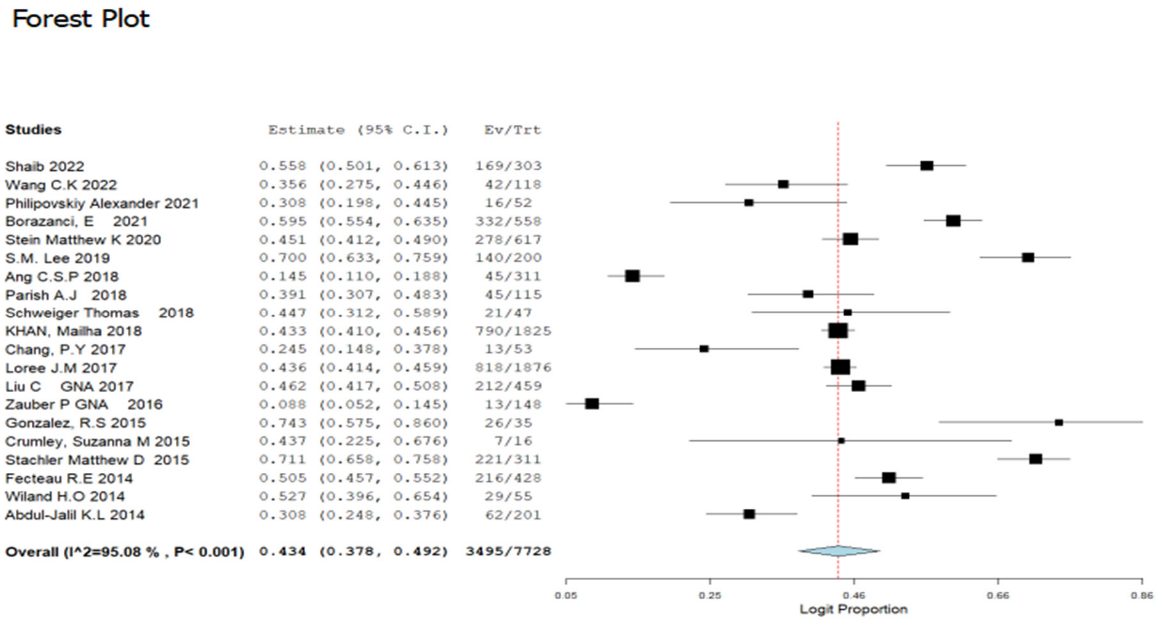

Figure S3. GNAS Mutation By Gender. Female Gender.

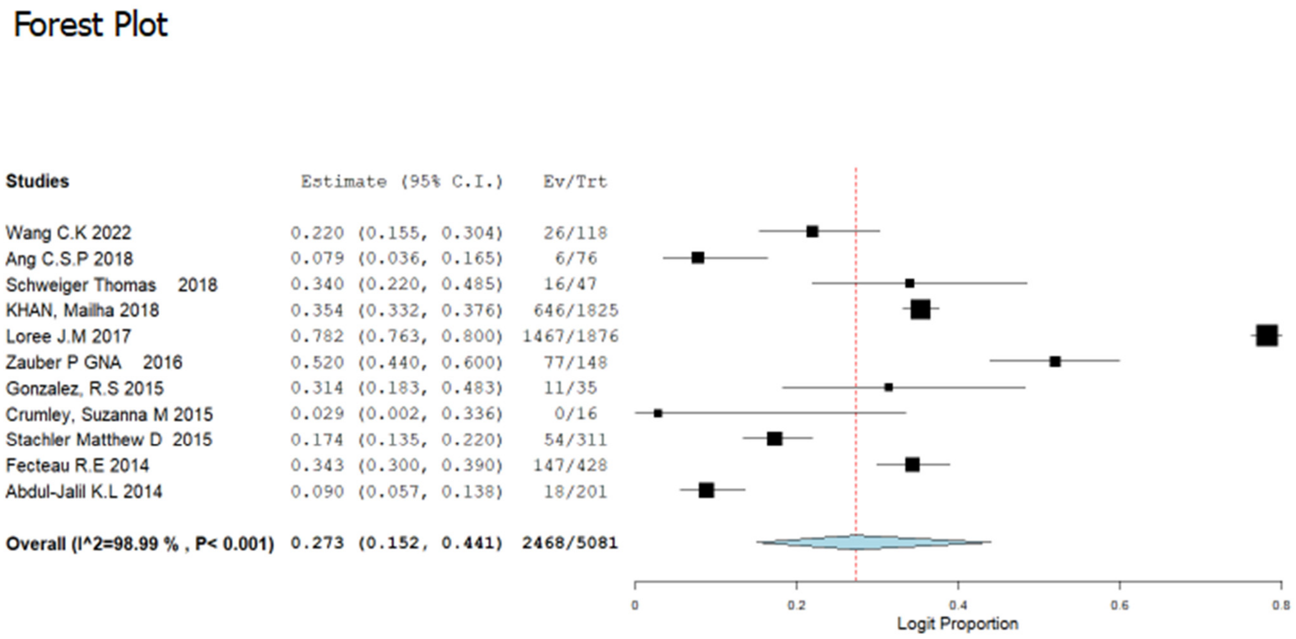

Figure S4. Gnas Mutation by Tumour Stages. Early Stage.

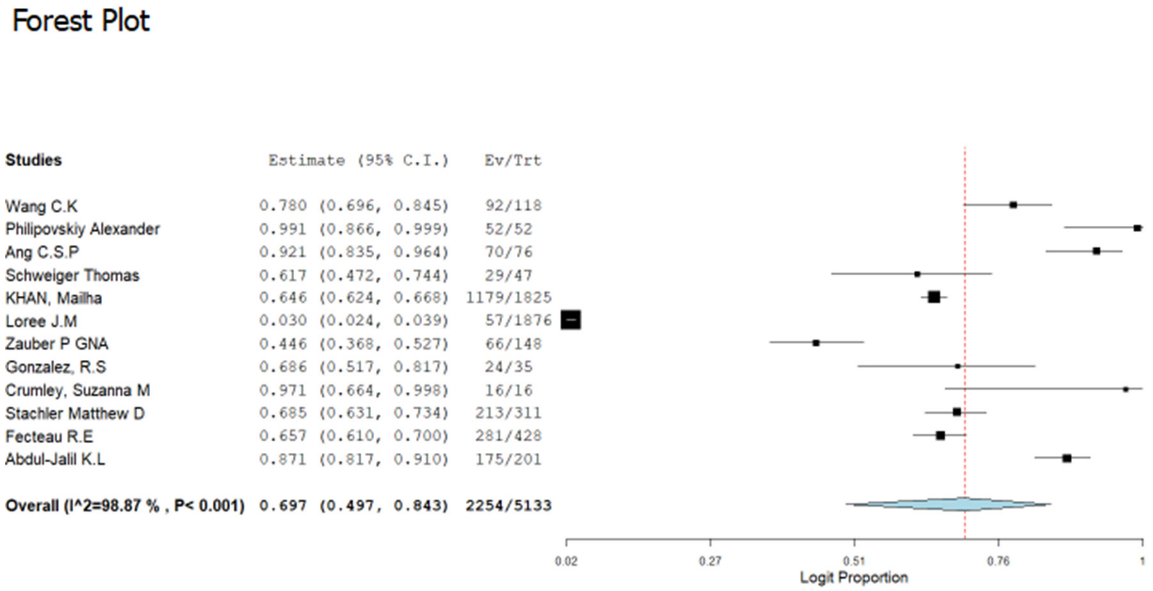

Figure S5. GNAS mutation by the Late tumour stage.

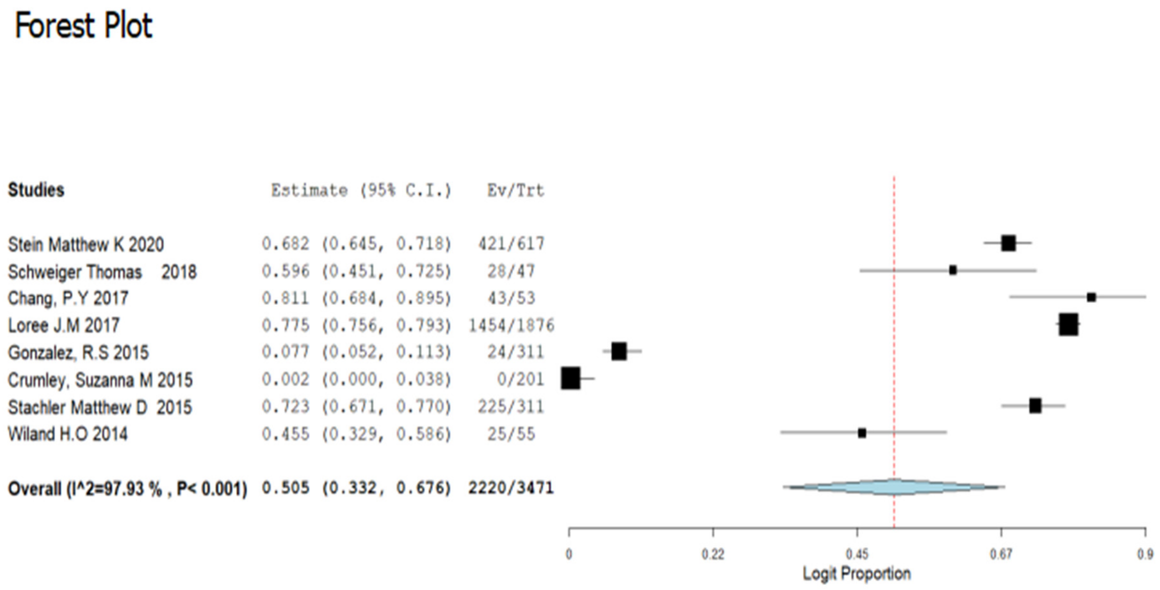

Figure S6. GNAS mutation by the Well tumour grade.

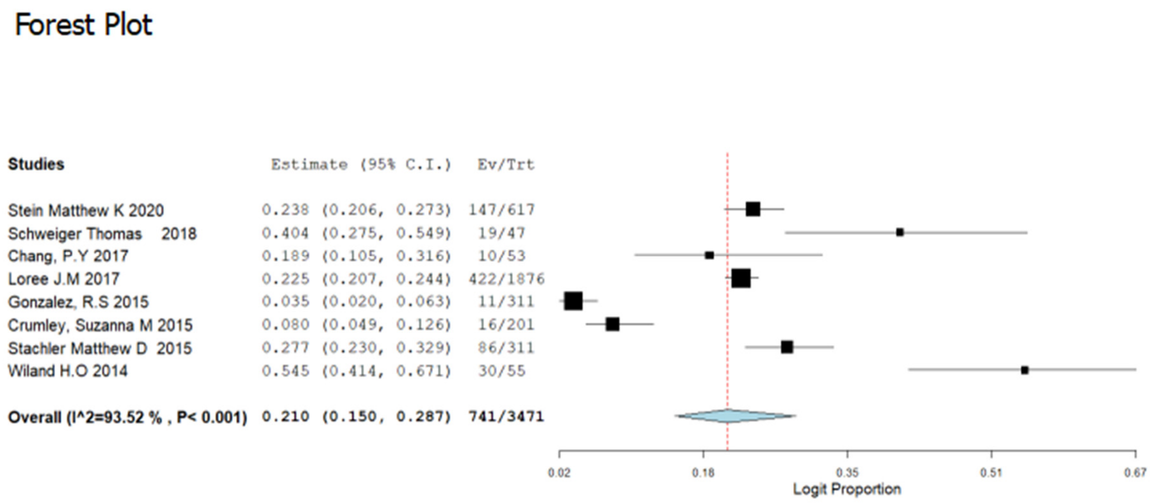

Figure S7. GNAS mutation by the Well tumour grade.

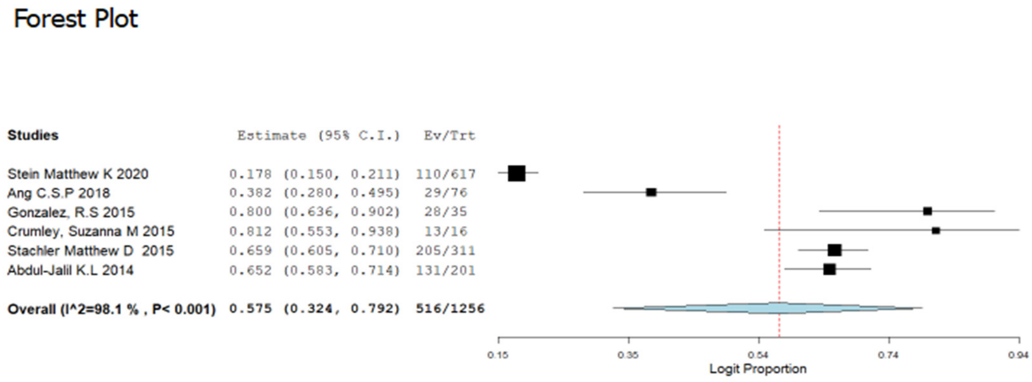

Figure S8. GNAS mutation by the Well tumour grade.

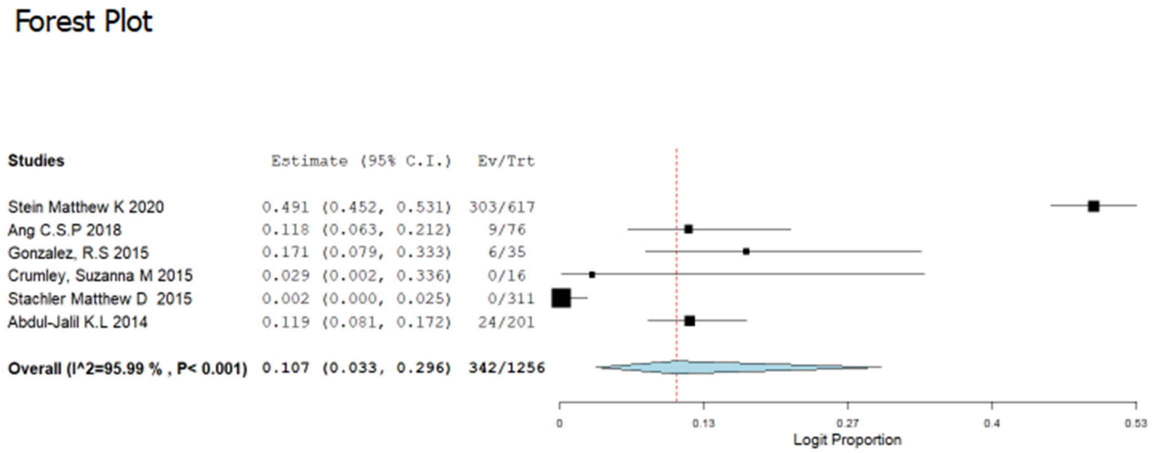

Figure S9. GNAS mutation by the Moderate tumour grade.

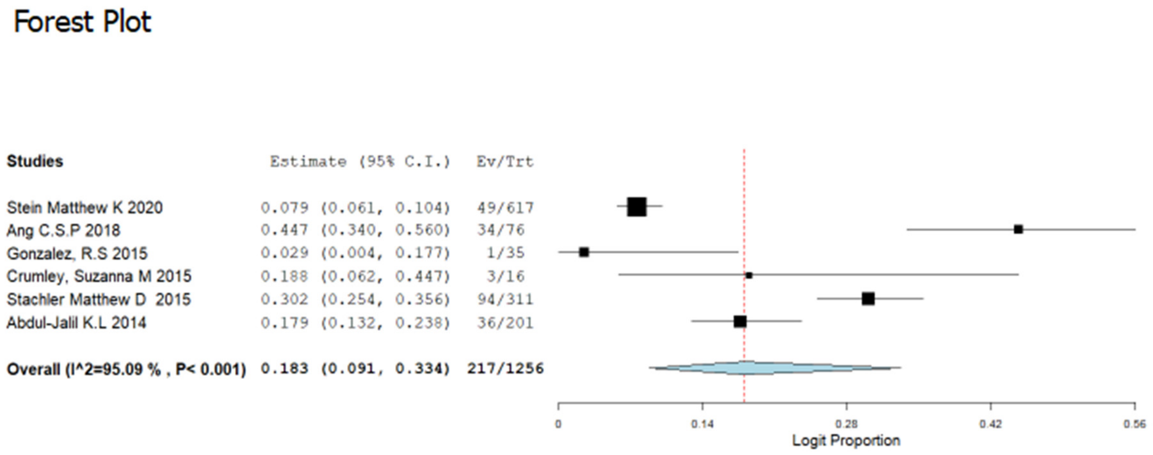

Figure S10. GNAS mutation by the Poor tumour gradeGNASGRADE-POOR.
