# Supplementary material for: A GNAS Gene Mutation’s Independent Expression in the Growth of Colorectal Cancer: A Systematic Review and Meta-Analysis
Source: Cancers (Basel). 2022 Nov 8;14(22):5480. doi: 10.3390/cancers14225480 (PMC9688108; doi:10.3390/cancers14225480)
Supplement: Supplementary file 1 [file cancers-14-05480-s001.zip › Table S2. JBI file.pdf]

Study name		Checklist*									Overall
		1	2	3	4	5	6	7	8	9	
1	Shaib 2022	Yes	No	Yes	Yes	Yes	Yes	Yes	Yes	unclear	7
2	Wang C.K 2022	Yes	No	Yes	No	Yes	Yes	Yes	Yes	Yes	7
3	Philipovskiy Alexander 2021	Yes	No	Yes	No	Yes	Yes	Yes	Yes	Yes	7
4	Borazanci, E 2021	Yes	No	Yes	Yes	Yes	Yes	Yes	Yes	Yes	8
5	Stein Matthew K 2020	Yes	No	Yes	Yes	Yes	Yes	Yes	Yes	Yes	8
6	S.M. Lee 2019	Yes	No	Yes	Yes	Yes	Yes	Yes	Yes	Yes	8
7	Tokunga R 2019	Yes	No	Yes	NO	Yes	Yes	Yes	Yes	Yes	7
8	Ang C.S.P 2018	Yes	No	Yes	Yes	Yes	Yes	Yes	Yes	Unclear	7
9	Parish A.J 2018	Yes	No	Yes	Yes	Yes	Yes	Yes	Yes	Yes	8
10	Schweiger Thomas 2018	Yes	No	Yes	No	Yes	Yes	Yes	Yes	Yes	7
11	KHAN, Mailha 2018	Yes	No	Yes	Yes	Yes	Yes	Yes	Yes	Yes	8
12	Lee, Hyunsu 2017	Yes	Yes	Yes	Yes	Yes	Yes	Yes	Yes	Yes	9
13	Chang, P.Y 2017	Yes	No	Yes	Yes	Yes	Yes	Yes	Yes	Yes	8
14	Loree J.M 2017	Yes	No	Yes	Yes	Yes	Yes	Yes	Yes	Yes	8
15	Liu C 2017	Yes	No	Yes	Yes	Yes	Yes	Yes	Yes	Yes	8
16	Lee Sung Hak 2017	Yes	Yes	Yes	Yes	Yes	Yes	Yes	Yes	Yes	9
17	Zauber P 2016	Yes	No	Yes	Yes	Yes	Yes	Yes	Yes	Yes	8
18	Jauhri M 2016	Yes	No	Yes	Yes	Yes	Yes	Yes	Yes	Yes	8
19	Gonzalez, R.S 2015	Yes	No	Yes	Yes	Yes	Yes	Yes	Yes	Yes	8
20	Crumley, Suzanna M 2015	Yes	No	Yes	Yes	Yes	Yes	Yes	Yes	Yes	8
21	Stachler Matthew D 2015	Yes	No	Yes	Yes	Yes	Yes	Yes	Yes	Yes	8
22	Alakus Hakan 2014	Yes	No	Yes	Yes	Yes	Yes	Yes	Yes	Yes	8
23	Fecteau R.E 2014	Yes	No	Yes	Yes	Yes	Yes	Yes	Yes	Yes	8
24	Walther B.M 2014	Yes	No	Yes	Yes	Yes	Yes	Yes	Yes	Yes	8
25	Wiland H.O 2014	Yes	No	Yes	Yes	Yes	Yes	Yes	Yes	Yes	8
26	Abdul-Jalil K.L 2014	Yes	No	Yes	Yes	Yes	Yes	Yes	Yes	Yes	8
27	Nishikawa G 2013	Yes	No	Yes	Yes	Yes	Yes	Yes	Yes	Yes	8
28	Yamada M 2012	Yes	No	Yes	Yes	Yes	Yes	Yes	Yes	Yes	7
29	Idziaszczyk S. 2010	Yes	No	Yes	Yes	Yes	Yes	Yes	Yes	Yes	8
30	Lamba S 2009	Yes	No	Yes	Yes	Yes	Yes	Yes	Yes	Yes	8

SUPPLEMENTARY FILE S3: The quality of the 30 included studies

* **1.** Appropriate sampling frame to address target population, **2.** Appropriate sampling way of study participants, **3.** Adequate sample size, **4.** Detail description of study participants and settings, **5.** Data analysis with sufficient coverage of identified sample, **6.** Use of valid methods to identify the condition, **7.** Standard, reliable way of measurement of condition for all participants, **8.** Availability of appropriate statistical analysis, **9.** Adequate response rate and management of low response rate

Quality of included studies by JBI critical appraisal checklist for studies reporting prevalence data
