# Supplementary material for: A GNAS Gene Mutation’s Independent Expression in the Growth of Colorectal Cancer: A Systematic Review and Meta-Analysis
Source: Cancers (Basel). 2022 Nov 8;14(22):5480. doi: 10.3390/cancers14225480 (PMC9688108; doi:10.3390/cancers14225480)
Supplement: Supplementary file 1 [file cancers-14-05480-s001.zip › Text S1. Search Strategic File (SSF).pdf]

SEARCH STRATEGY

WEB OF SCIENCE (N=142)
((ALL=colorectal cancer OR ALL=colorectal carcinoma OR ALL=colon cancer OR ALL=colon carcinoma OR ALL=colorectal neoplasm OR ALL=colon neoplasm OR ALL=metastatic colon cancer OR ALL=metastatic colorectal cancer OR ALL=colorectal tumour OR ALL=colon tumour OR ALL=rectum OR ALL=CRC OR ALL=mCRC) AND (ALL=GNAS OR ALL=GNAQ OR ALL=gnas1 OR ALL=G-NAS OR ALL=G-NAQ OR ALL=G-NA11 OR ALL=GNA))

PUBMED (N=54)
("colorectal cancer"[Title/Abstract] OR "colorectal carcinoma"[Title/Abstract] OR "colon cancer"[Title/Abstract] OR "colon carcinoma"[Title/Abstract] OR "colorectal neoplasm"[Title/Abstract] OR "colon neoplasm"[Title/Abstract] OR "metastatic colon cancer"[Title/Abstract] OR "metastatic colorectal cancer"[Title/Abstract] OR "colorectal tumour"[Title/Abstract] OR "colon tumour"[Title/Abstract] OR "rectum"[Title/Abstract] OR "CRC"[Title/Abstract] OR "mCRC"[Title/Abstract]) AND ("GNAS"[Title/Abstract] OR "GNAQ"[Title/Abstract] OR "GNA11"[Title/Abstract] OR "GNA"[Title/Abstract])

SCOPUS FORMAT 58 (N=58)
TITLE-ABS ("colorectal cancer" OR "colorectal carcinoma" OR "colon cancer" OR "colon carcinoma" OR "colorectal neoplasm" OR "colon neoplasm" OR "metastatic colon cancer" OR "metastatic colorectal cancer" OR "rectum" OR "CRC" OR "mCRC" OR "colorectal tumour" OR "colon tumour") AND TITLE-ABS ("GNAS" OR "GNAQ" OR "GNA11" OR "GNA")

GOOGLE SCHOLAR (N=5)
allintitle:("colorectal cancer" OR "colorectal carcinoma" OR "colon cancer" OR "colon carcinoma" OR "colorectal neoplasm" OR "colorectal tumour" OR "colon tumour") ("GNAS" OR "GNAQ" OR "GNA11" OR "GNA")

SCIENCE DIRECT (N=12)
("colorectal cancer" OR "colorectal carcinoma" OR "colon cancer" OR "colon carcinoma" OR "colorectal tumour" OR "colon tumour") ("GNAS" OR "GNAQ" OR "GNA11" OR "GNA")
